# Comparison of 180° anti-reflux mucosectomy versus 270° anti-reflux mucosectomy for treatment of refractory gastroesophageal reflux disease: a retrospective study

**DOI:** 10.1007/s00464-021-08857-5

**Published:** 2021-11-15

**Authors:** Xinyi Yang, Jiacheng Tan, Yang Liu, Yadong Feng, Ruihua Shi

**Affiliations:** Department of Gastroenterology, Zhongda Hospital, School of Medicine, Southeast University, Dingjiaqiao street-87, Nanjing, 210009 Jiangsu China

**Keywords:** Refractory gastroesophageal reflux disease, Endoscopic treatment, Anti-reflux mucosectomy, Dysphagia

## Abstract

**Background:**

Anti-reflux mucosectomy (ARMS) is a novel endoscopic treatment for refractory gastroesophageal reflux disease (rGERD). Several studies have validated its safety and effectiveness, but postoperative dysphagia remains in concern. Since the influence of different resection ranges on efficacy and complications of ARMS has rarely been studied, this study aimed to compare outcomes of 180°ARMS and 270°ARMS in treatment of rGERD.

**Methods:**

This study was conducted from August 2017 to September 2020. 39 eligible patients underwent either 180° ARMS or 270° ARMS and followed up at 6 months postoperation. Primary outcome measure was assessed by Gastroesophageal Reflux Disease Questionnaire (GERD-Q). Secondary outcomes included quality of life, PPI use, gastroesophageal flap valve grade, presence of reflux esophagitis, acid exposure time (AET), distal contractile integral (DCI), and integrated relaxation pressure (IRP) measured by high-resolution manometry (HRM) and complication rate. Per-protocol analysis was performed.

**Results:**

Among 39 patients, 18 underwent 180° ARMS, while 21 underwent 270° ARMS. At postoperative 6 months follow-up period, primary outcome showed no significant difference between two groups (*p* = 0.34). Similarly, no significant difference was demonstrated between groups regarding most secondary outcomes except for fewer complaints of newly dysphagia in 180° ARMS group. No other serious complications were observed in both groups.

**Conclusion:**

Although 180° ARMS and 270° ARMS could be equally effective for treatment of rGERD, 180° ARMS might be more recommended due to lower incidence of newly post-procedural dysphagia.

**Supplementary Information:**

The online version contains supplementary material available at 10.1007/s00464-021-08857-5.

Gastroesophageal reflux disease (GERD) is one of the most common gastrointestinal disorders, defined as a condition when reflux of gastric contents cause troublesome symptoms and/or complications [1, 2].

Management of GERD commonly begins with lifestyle modification and medical therapy. Acid-suppressive therapy, especially proton-pump inhibitor (PPI) therapy, acts as the mainstay of therapy. Although highly effective for healing reflux esophagitis, PPIs are less effective in controlling reflux symptoms in up to 30% patients with GERD despite optimized PPI therapy [3]. Moreover, it has been reported that long-term use of PPIs is associated with various adverse effects, for instance infectious complications, osteoporosis, and decrease in micronutrient absorption [5, 6].

Persistent and troublesome GERD symptoms nonresponsive partially or completely after at least 8 weeks of a standard-dose PPI therapy may be termed as refractory GERD (rGERD) [4]. Treatments for patients with rGERD include anti-reflux surgery and endoscopic anti-reflux treatments. Although gold standard treatment in such cases remains laparoscopic Nissen fundoplication (LNF), but acceptance of this treatment among patients is gradually declining in recent years owing to its invasive nature, high rate of reflux recurrence, and potential side effects such as abdominal bloating and dysphagia [7, 8]. Therefore, as alternative approaches, endoscopic anti-reflux treatments have been rapidly developed and have gained popularity among those who suffer from rGERD. Several endoscopic therapies have been developed in hope of narrowing treatment gap between PPIs therapy and need of anti-reflux surgery [9–11], including radiofrequency ablation (RFA) to lower esophageal sphincter (LES) [12, 13] and transoral incisionless fundoplication (TIF) [14, 15]. However, clinical application of endoscopic therapies above has not been widespread due to their high cost of proprietary devices. In addition, long-term efficacy of these endoscopic therapies has not been validated.

Anti-reflux mucosectomy (ARMS), a novel technique, was initially developed by Inoue et al. [16] based on standardized techniques of endoscopic mucosal resection (EMR) and endoscopic submucosal dissection (ESD). Aiming to restore a morphologically and functionally anti-reflux barrier, this mucosal resection technique augments constriction of gastric cardia by scar formation during mucosal healing. Excision of gastric mucosa containing oxyntic glands might result in decreased gastric acid secretion and contribute to anti-reflux mechanism of ARMS. Furthermore, advantages of non-requirement of any additional devices and artificial prostheses in situ make ARMS a promising distinct alternative for treatment of rGERD.

In the seminal study conducted by Inoue et al., reflux and GERD symptom have been well controlled by ARMS [16]. And, PPIs could be discontinued postoperatively in all patients. Subsequently, several studies validated effectiveness and safety of ARMS in treatment of rGERD [17, 18]. However, quality evidence and follow-up data are still scarce. It is worth mentioning that dysphagia, as a procedure-associated complication, was reported in all of the published articles above.

Gastroesophageal valve flap (GEFV) is a functional valve flap composed of His angle and mucosa of greater curvature to increase gastroesophageal pressure gradient. A patent valve flap can effectively prevent reflux. Acid pocket refers to an unbuffered, strong acid secretory region in proximal stomach near gastroesophageal junction (GEJ). This area is formed after meals and is the source of acid reflux toward esophagus. When patient has esophageal hiatal hernia and/or weak lower esophageal sphincter, acid pocket invades gastroesophageal junction and squamous epithelium of distal esophagus is exposed to strong acid, resulting in mucosal injury and reflux symptoms. Acid pocket is considered to be an important target for pharmacology and treatment of GERD. In Inoue’s study, 10 patients with rGERD were included, first 2 cases underwent circumferential resection and remaining 8 underwent 180°–270° resection. Our center initially selected 270° for resection based on preserving patients' normal GEFV while removing as much acid pocket as possible. The first three patients had short-term postoperative dysphagia, and then 180° ARMS was selected. After that, patients were randomly divided into two groups. The relevant data before and after operation were recorded; surgical effects and postoperative complications of 180° ARMS and 270° ARMS were studied retrospectively.

To date, there have been few studies determining the influence of different resection ranges on efficacy and adverse effects of ARMS. In this study, we aimed to compare clinical outcomes of two different resection ranges of 180° ARMS and 270° ARMS in treatment of rGERD.

## Methods

### Study design and patient selection

This was a single-center, retrospective study with patients who underwent 180° ARMS or 270° ARMS from August 2017 to September 2020. Study protocol was approved by institutional review board of Zhongda Hospital (Approval Number: 2020ZDSYLL253-P01). Written informed consent was obtained from all participants. Detailed inclusion and exclusion criteria for participants are listed in Supplementary Table S1.

### Preoperative assessments

Patients were admitted at least 2 days before ARMS procedure for a range of preoperative assessments. Medical history (especially details of PPI use) was obtained and routine laboratory tests were performed.

Reflux symptoms were evaluated with Gastroesophageal Reflux Disease Questionnaire (GERD-Q) (Supplementary Table S2) and quality of life was assessed with Gastroesophageal Reflux Disease-Health-Related Quality of Life scale (GERD-HRQL) (Supplementary Table S3). GERD-Q score ranged from 0 to 18, with a score ≥ 8 being highly suggestive of GERD [19]. GERD-HRQL scale measured disease-specific quality of life by scoring 10 items, which ranged from 0 to 50, with higher scores indicating more severe symptoms [20].

Preoperative upper endoscopy was performed to assess grade of GEFV based on Hill classification (a grading system developed by Hill et al. in which geometry of flap valve was scored from I to IV according to its endoscopic appearance) [21]. Patients with large hiatal hernia > 2 cm or severe reflux esophagitis of grade C or D according to Los Angeles classification [22] were ruled out from this study.

Ambulatory reflux monitoring was performed continuously for 24 h using Orion portable pH monitor (Medical Measurements Systems Enschede, The Netherlands). Acid exposure time (AET, percentage of time with pH < 4 over measurement duration) is the most commonly used parameter to evaluate esophageal acid burden. Distal esophageal acid exposure was considered pathological when total AET was more than 6% in 24-h pH monitoring [23].

All patients underwent esophageal high-resolution manometry (HRM) preoperatively to exclude esophageal motility disorders such as achalasia, using Solar GI gastrointestinal dynamic inspection system (Medical Measurements Systems, Enschede, The Netherlands). Esophageal body contraction vigor and EGJ relaxation were measured using distal contractile integral (DCI) and integrated relaxation pressure (IRP), respectively [24, 25].

Oral drug treatment is still recommended for patients with negative evaluations preoperatively. If they have not taken psychotropic drugs before, appropriate consideration can be given to add anti-anxiety and anti-depression treatment.

### Anti-reflux mucosectomy (ARMS) procedure

**Fig. 1:**
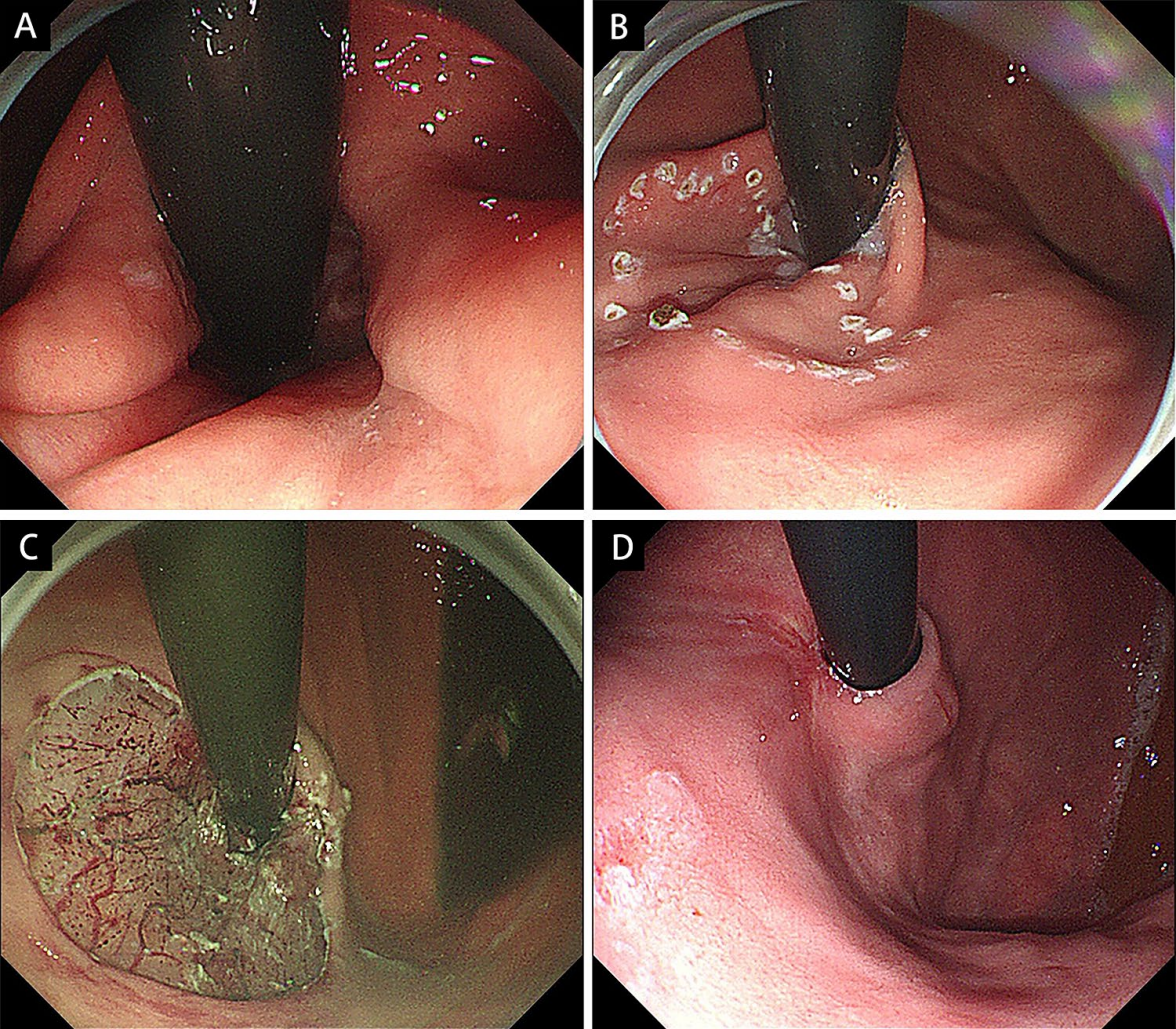
Endoscopic follow-up of 180° anti-reflux mucosectomy (ARMS). **A** Preoperative retroflexed view. **B** Marking of expected resection area.** C** Approximately hemi-circumferential esophagogastric junction (EGJ) mucosa was resected. **D** 6-month postoperative retroflexed view

**Fig. 2:**
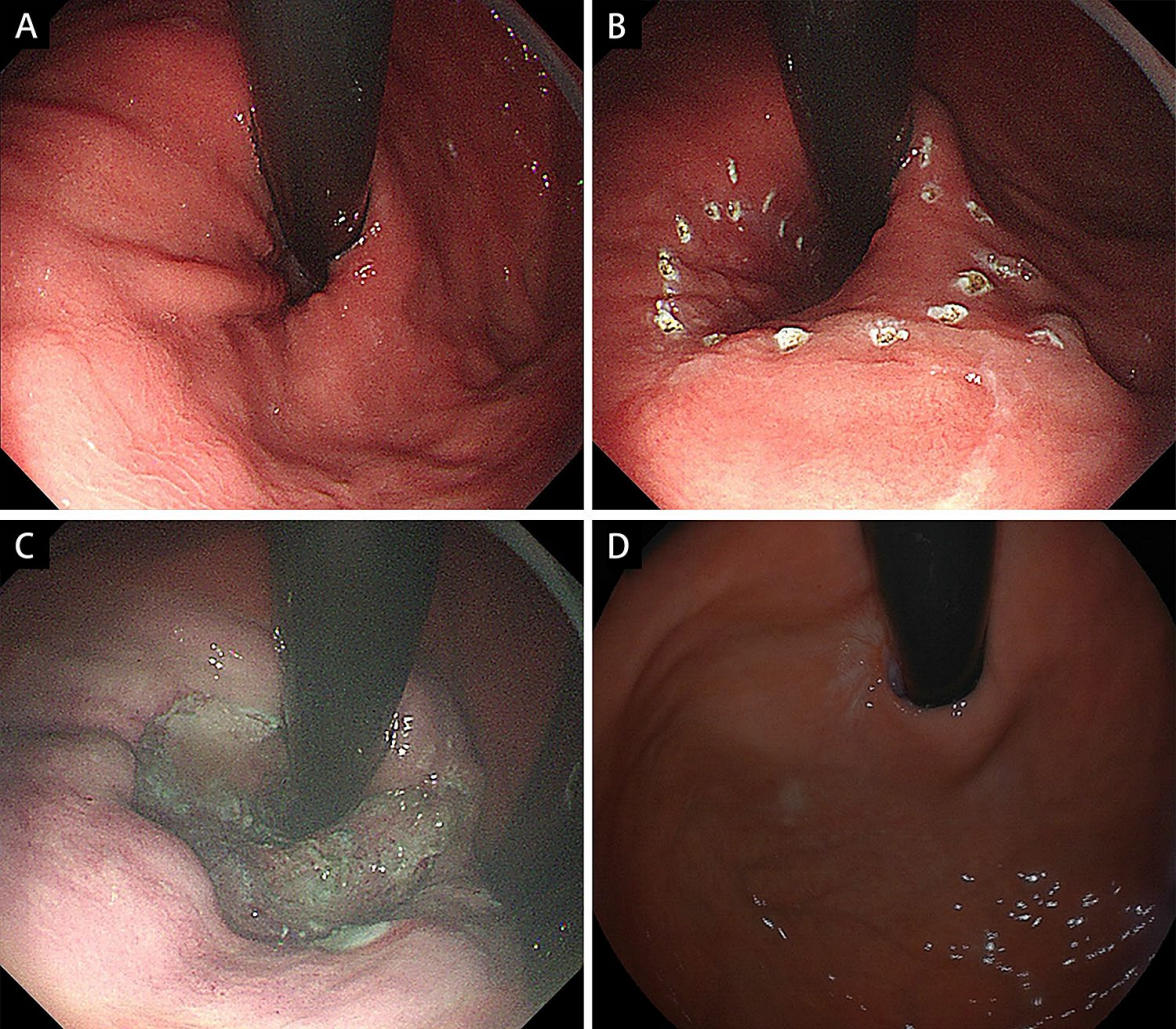
Endoscopic follow-up of 270° anti-reflux mucosectomy (ARMS). **A** Preoperative retroflexed view.** B** Marking of expected resection area. **C** Mucosal resection of approximately three fourths of circumference of esophagogastric junction (EGJ) was performed. **D** 6-month postoperative retroflexed view

ARMS procedures were performed by a single expert endoscopist, based on standardized techniques of ESD. A high-definition gastroscope (GIF-HQ290, Olympus, Tokyo, Japan) equipped with a transparent distal cap was applied for the procedure, using only carbon dioxide for insufflation. The mucosal resection at least 3 cm in length (1 cm in esophagus and 2 cm in stomach) was conducted along lesser curvature of stomach and gastric sling fiber at greater curvature side was preserved. Preoperative, intraoperative, and 6-month postoperative endoscopic retroflexed views of EGJ in two ranges were demonstrated in Figs. 1 and 2.

The scheduled margin of mucosal resection area was marked around EGJ with spotty cautery (Figs. 1B and 2B) using Dual knife (KD-650L, Olympus, Tokyo, Japan) connected with high-frequency generator (VIO300D, ERBE Elektromedizin, Tübingen, Germany) in forced coagulation mode (30 W effect 2 in esophagus and 50 W effect 2 in stomach). A mixture of normal saline, diluted epinephrine (1:10,000), and indigo carmine was then injected into submucosal layer using a 25-gage needle with a 4-mm tip. Marginal incision was performed along markings applying Dual knife with Endocut Q current 3–2–4. Submucosal dissection and complete mucosal resection were accomplished with Dual knife in forced coagulation mode (40 W effect 2 in esophagus and 50 W effect 2 in stomach). In procedure of 180° and 270° ARMS, crescentic mucosal resection of 50% and 75% circumference of EGJ was performed, respectively (Figs. 1C and 2C).

Hemostasis was carried out using a Coagrasper (FD-410LR, Olympus, Tokyo, Japan) with forced coagulation mode at 50–60 W effect 2. Fibrin sealant (RAAS, Shanghai, China) was then sprayed and completely covered mucosal defect. For those with intraoperative muscularis propria injury, which was difficult to close only with fibrin sealant, hemostatic clips (MICRO-TECH, Nanjing, China) were applied to prevent perforation or bleeding.

### Postoperative management and follow-up

Double-dose PPIs and antibiotics were administered intravenously to all patients after ARMS. Patients had no oral intake for 24 h after ARMS and then initiated a liquid diet next day if no complications were noted. In case of obvious nausea and/or pain, antiemetic medication and/or analgesic were administered. Emergency endoscopy was performed in patients with severe abdominal pain or other signs of bleeding or perforation. At discharge, patients were advised to take a soft diet for 2 weeks. The double-dose PPIs were prescribed for subsequent 2 months and then taken only on demand. Mucosal protective agents (polaprezine, teprenone) and prokinetic agents (mosapride) were also prescribed for 1–2 months after ARMS according to individual recovery.

All patients were scheduled for 6-month follow-up visits after ARMS. Subjective feelings of patients including symptom severity and quality of life were assessed quantitatively using GERD-Q and GERD-HRQL. The frequency and dose of PPI use were recorded as well. Upper endoscopy, esophageal 24-h pH monitoring, and HRM were performed according to study protocol.

### Assessment of study outcomes

The primary outcome of this study was assessed by GERD-Q. A total of 8 secondary outcome measures were assessed at baseline and follow-up visits, including quality of life, PPI use, gastroesophageal flap valve grade, presence of reflux esophagitis, acid exposure time (AET), distal contractile integral (DCI), and integrated relaxation pressure (IRP) measured by high-resolution manometry (HRM) and complication rate. Serious complications were defined as adverse events requiring hospitalization and additional endoscopic or surgical intervention.

### Statistical analysis

All statistical analysis was performed using SPSS 23.0 statistics software (SPSS Inc, Chicago, Illinois, USA). Study data were obtained from patients who accomplished follow-up and were summarized by means of standard descriptive statistics. A per-protocol analysis was performed.

Continuous variables were presented as mean ± standard deviation (SD) or median (range) depended on the distribution. Categorical variables were expressed in way of frequencies and percentages unless stated otherwise. For continuous variables, comparisons of parametric data were conducted using Student’s t-test, whereas differences of non-parametric data were assessed with Wilcoxon signed-rank test or Mann–Whitney *U* test. Fisher’s Exact test was applied for categorical variables. In order to compare GEFV grade, grades I, II, III, and IV were transformed into score of 1, 2, 3, and 4 points, respectively. Two-sided *p* values < 0.05 were considered statistically significant.

## Results

### Enrollment and patient characteristics

From August 2017 to September 2020, 45 patients with rGERD were assessed for eligibility, among whom 6 patients were excluded ultimately (3 were diagnosed with achalasia, 2 did not consent to endoscopic surgery and 1 declined preoperative 24-h pH monitoring). Hence, 39 patients who provided informed consent were recruited in this study: 18 underwent 180° ARMS and 21 underwent 270° ARMS. All cases went follow-up at 6 months. Baseline characteristics were similar between two groups, except for a longer procedure time in 270° ARMS group (Table 1). Furthermore, there was no significant difference between groups regarding to results of preoperative assessments (Table 2).

**Table 1:** Baseline characteristics

Variables	180° ARMS (*n* = 18)	270° ARMS (*n* = 21)	*p* Value
Age, mean ± SD, y	53.11 ± 7.62	51.33 ± 12.39	0.588
Sex, *n* (%)			> 0.99
Male	12 (66.7)	16 (76.2)	
Female	6 (33.3)	5 (23.8)	
BMI, mean ± SD, kg/m^2^	23.82 ± 2.87	24.01 ± 3.65	0.859
Symptom duration, median (range), y	1.5 (1.00–10.75)	3(1.00–4.50)	0.606
PPI use, *n* (%)			0.900
Occasional	3 (16.7)	0	
Daily single dose	9 (50.0)	9 (42.8)	
Daily double dose	6 (33.3)	12 (57.2)	
Procedure time, median (range), min	35 (29–58)	55.5 (43–70)	< 0.01

*ARMS* anti-reflux mucosectomy, *SD* standard deviation, *BMI* body mass index, *PPI* proton-pump inhibitor

**Table 2:** Preoperative assessments

Variables	180° ARMS (*n* = 18)	270° ARMS (*n* = 21)	*p* value
GERD-Q score, mean ± SD	11.50 ± 2.48	11.14 ± 2.37	0.649
GERD-HRQL score, mean ± SD	20.6 ± 5.0	19.5 ± 3.7	0.54
GEFV grade, median (range)	3 (2–4)	3 (2–4)	0.46
Hiatal hernia, *n* (%)	2 (11.1)	4 (19.0)	0.667
Reflux esophagitis, *n* (%)			0.069
None	4 (22.2)	3 (14.3)	
Grade A	11 (61.1)	7 (33.3)	
Grade B	3 (16.7)	11 (52.4)	
AET, mean ± SD, %	18.5 ± 7.5	19.4 ± 7.2	0.78
DCI, mean ± SD, mmHg*s*cm	626.3 ± 102.1	568.6 ± 97.8	0.16
IRP, mean ± SD, mmHg	5.1 ± 1.0	4.6 ± 0.8	0.17

*ARMS* anti-reflux mucosectomy, *GERD-Q* gastroesophageal reflux disease questionnaire, *SD*standard deviation, *GERD-HRQL* gastroesophageal reflux disease-health-related quality of life, *GEFV* gastroesophageal flap valve, *AET* acid exposure time, *DCI* distal contractile integral, *IRP* integrated relaxation pressure

### Primary outcome

The adverse symptoms such as reflux and heartburn were significantly improved after ARMS. The average GERD-Q score was 11.38 ± 40 (*n* = 39), which decreased to 6.60 ± 63 after 6 months of operation (*n* = 39, *p* < 0.01). There were significant differences in results of GERD-Q scores before and after operation in both two groups (*p* < 0.01). However, there was none found in GERD-Q scores between two groups at follow-up time point (*p* > 0.05) (Tables 3,4).

**Table 3:** Comparison of clinical outcomes between groups

	180° ARMS (*n* = 18)	270° ARMS (*n* = 21)	*p* Value
Primary outcome			
GERD-Q score, mean ± SD	6.9 ± 2.2	6.1 ± 1.8	0.34
Secondary outcomes			
GERD-HRQL score, mean ± SD	8.7 ± 3.6	10.0 ± 3.9	0.39
PPI use, *n* (%)			0.53
Discontinued	9 (50.0)	14 (66.67)	
Reduced	5 (27.8)	4 (19.04)	
Maintained	4 (22.2)	3 (14.29)	
GEFV grade, median (range)	1.5 (1–3)	1 (1–3)	0.60
Reflux esophagitis, n (%)			0.71
None	16 (88.88)	14 (66.67)	
Grade A	1 (5.56)	4 (19.04)	
Grade B	1(5.56)	3 (14.29)	
AET, mean ± SD, %	7.9 ± 4.6	6.8 ± 5.2	0.56
DCI, mean ± SD, mmHg*s*cm	781.2 ± 122.6	841.3 ± 108.1	0.20
IRP, mean ± SD, mmHg	7.9 ± 1.7	9.0 ± 2.2	0.17
Postoperative complications, *n* (%)			
Dysphagia (newly occurred)	2	7	0.04
Dysphagia (treated with repeated dilations)	1 (5.56)	4 (19.04)	0.349
Bleeding	0	1 (4.76)	0.46

*ARMS* anti-reflux mucosectomy, *SD* standard deviation, *GERD-Q* gastroesophageal reflux disease questionnaire, *GERD-HRQL* gastroesophageal reflux disease-health-related quality of life, *PPI* proton-pump inhibitor, *GEFV* gastroesophageal flap valve, *AET* acid exposure time, *DCI* distal contractile integral, *IRP* integrated relaxation pressure

**Table 4:** Comparison of clinical outcomes in two groups pre- and post-operatively

Variables	180° ARMS (*n* = 18)			270° ARMS (*n* = 21)		
	Pre	Post	*p* value	Pre	Post	*p* value
GERD-Q score, mean ± SD	11.50 ± 2.48	6.9 ± 2.2	< 0.01	11.14 ± 2.37	6.1 ± 1.8	< 0.01
GERD-HRQL score, mean ± SD	20.6 ± 5.0	8.7 ± 3.6	< 0.01	19.5 ± 3.7	10.0 ± 3.9	< 0.01
AET, mean ± SD, %	18.5 ± 7.5	7.9 ± 4.6	< 0.01	19.4 ± 7.2	6.8 ± 5.2	< 0.01
DCI, mean ± SD, mmHg*s*cm	626.3 ± 102.1	781.2 ± 122.6	< 0.01	568.6 ± 97.8	841.3 ± 108.1	< 0.01
IRP, mean ± SD, mmHg	5.1 ± 1.0	7.9 ± 1.7	< 0.01	4.6 ± 0.8	9.0 ± 2.2	< 0.01

*ARMS* anti-reflux mucosectomy, *SD* standard deviation, *GERD-Q* gastroesophageal reflux disease questionnaire, *GERD-HRQL* gastroesophageal reflux disease-health-related quality of life, *AET* acid exposure time, *DCI* distal contractile integral, *IRP* integrated relaxation pressure

### Secondary outcomes

Secondary outcomes were assessed at baseline and at 6 months after treatment. Analysis of results showed no significant difference between two groups.

As depicted in Table 3, there was no significant difference between groups in terms of GERD-HRQL scores (*p* = 0.39), changes in PPI use (*p* = 0.53), and AET (*p* = 0.56). However, significant difference was noted in results of AET before and after operation in both two groups (*p* < 0.01). At baseline, almost all patients in both groups were on daily PPI medications. After the operation, 58.97% (23/39, 9 in 180°ARMS group and 14 in 270°ARMS group) patients discontinued their use of PPIs and a reduction in dose or frequency of PPI was reported as 5 patients treated with 180° ARMS and 4 patients treated with 270° ARMS. Only 7 patients (4 in 180° ARMS group and 3 in 270° ARMS group) remained on preoperative PPI usage.

GEFV was restored and well defined after both ranges of ARMS (Figs. 1D and 2D). The endoscopic findings demonstrated a similar distribution of GEFV grade among two groups. The median flap valve grade was 1.5 in 180° ARMS group and 1 in 270° ARMS group (*p* = 0.60). In addition, presence of mild reflux esophagitis (Los Angeles Grade A/B) was decreased from 77.8% (14/18) to 11.12% (2/18) after 180° ARMS procedure and 85.7% (18/21) to 33.33% (7/21) in patients treated with 270° ARMS.

Preoperative and postoperative statistical P values of DCI and IRP in both two groups were < 0.01 (Table 4). However, there were no significant differences noted between two groups in terms of DCI and IRP on 6 months postoperatively. The mean DCI according to 6-month HRM findings was 781.2 mmHg*s*cm in 180° ARMS group and 841.3 mmHg*s*cm in 270° ARMS group (*p* = 0.20), while the mean IRP was 7.9 mmHg in patients who underwent 180° ARMS and 9.0 mmHg in patients who underwent 270° ARMS (*p* = 0.17).

As expected, after procedure, 9 patients suffered from dysphagia of varying degrees (2 in 180° ARMS group and 7 in 270° ARMS group, *p* = 0.04). Four patients with mild dysphagia gradually recovered to normal swallowing over time, while other 5 patients (1 in 180° ARMS group and 4 in 270° ARMS group) were treated with repeated Savary-Gilliard bougie dilations due to esophageal stenosis. Moreover, postoperative bleeding occurred in 1 patient treated with 270° ARMS, which was then successfully managed by endoscopic hemostasis. No other serious procedure-related complications were observed in both groups.

## Discussion

Our retrospective study compared clinical outcomes of different ranges in ARMS procedure and demonstrated that both ranges of ARMS could rebuild an anatomically and functionally anti-reflux barrier. Similar short-term outcomes of two ranges of ARMS suggested that hemi-circumferential mucosal resection might be enough to create a tight and robust anti-reflux barrier; however, a more realistic conclusion can be drawn only with larger samples and longer follow-up. Meanwhile, in comparison with 270° ARMS, 180° ARMS might have advantage of fewer complaints about newly dysphagia according to present findings.

Moreover, overall efficacy of ARMS in this study was similar to reported results in other retrospective and some uncontrolled prospective studies, which demonstrated symptom improvement rates of 68–88% and PPI discontinuation rates of 68% to 100% after 6 months or longer [17–19]. The improvement on distal esophageal acid exposure was not as significant as reported in pilot study by Inoue et al. [16] and a slightly higher proportion of normalized distal esophageal acid exposure at 6 months after treatment was accomplished in our study than those treated with TIF or PPI therapy [15].

Another advantage of this study was the regular and comprehensive follow-up schedule, consisting of subjective feelings measured by GERD-Q and GERD-HRQL scales, concrete PPI use, endoscopy, 24-h esophageal pH monitoring, and HRM. All these measures contribute to confirm both subjective and objective efficacy of ARMS as an endoscopic treatment alternative for patients with rGERD. Given the possible recurrence of GERD after treatment, which have already been reported after LNF and TIF in median-term and long-term studies [8, 14, 15], regular postoperative review after ARMS is deemed necessary and would be continued in our subsequent study.

Additional advantage of this study is that ARMS was completed on basis of ESD technique in all patients. Both ESD and multi-fragment EMR were applied in initial study by Inoue et al. [16], other two studies used cap-assisted EMR (EMR-C) or EMR with a band ligation device (EMR-L) [17, 18]. Moreover, complete excision of expected mucosa using ESD technique can provide intact pathological samples, which cannot be achieved with multi-fragment EMR. Furthermore, operative specimen can be utilized for research on pathogenesis of rGERD.

As major complication after ARMS, postoperative dysphagia is always a concern for both endoscopists and patients. Likewise, high incidence of dysphagia following anti-reflux surgery have been demonstrated in many studies [26–28]. The overall rate of postoperative dysphagia and need for esophageal dilatations were slightly higher in our study than in previous studies [29, 30]. One contributing mechanism behind might be excessive contraction caused by early postoperative changes (inflammation, fibrosis, and scar formation), which is reversible in most cases. Larger extent of mucosal resection causes more intense inflammation and scar formation leading to increased probability of postoperative stenosis, which accounted for higher incidence of newly dysphagia in 270° ARMS group [31, 32]. In addition, notable IRP elevation observed in post-ARMS patients may result in overcorrection of EGJ relaxation, which might impede bolus clearance and contribute to dysphagia [33]. It is worth mentioning that degree of scar formation, as key to restore the anti-reflux barrier, is significantly influenced by gastric acid [17]. Thus, the 2-month acid suppression therapy with double-dose PPIs and rational use of adjunct medications (mucosal protective agents and prokinetic agents) after ARMS are crucial and indispensable in reducing dysphagia.

Balloon or probe dilation can be used to treat postoperative dysphagia. Since the wound after operation is large and cannot be clipped with titanium clip, hence, our center is covering the wound with tissue glue or PGA biofilm to observe whether it can accelerate healing and reduce incidence of postoperative complications.

There are several limitations in this study. Firstly, limited sample size, short follow-up period, and lack of an appropriate control group treated with LNF (the current gold standard treatment for rGERD) which might limit the evidence to confirm efficacy of ARMS. As a new procedure still in preliminary and small-scale clinical practice, the initial clinical outcomes of ARMS are encouraging, but only with substantially larger cohort and longer clinical experience, more realistic evaluation could be obtained. Five-year follow-up data of included patients are being collected and will be reported. Meanwhile, further research on predictors of clinical response to ARMS with larger samples is ongoing. Moreover, our research team has recently initiated a randomized controlled trial on ARMS versus LNF including eligible patients with rGERD, in cooperation with department of general surgery. Preliminary results are expected to be reported in 2023 and may identify definite efficacy of ARMS in treatment of rGERD.

Another limitation of our study is that all ARMS procedures were performed by a single highly experienced endoscopist with expertise in esophageal and gastric ESD techniques. Although this is conducive to standardize results, it is unclear whether this technique would be equally effective and safe when performed by endoscopists with less-experience. Hence, multicenter studies involving multiple endoscopists are necessary to further validate its feasibility and efficacy.

Additionally, due to limitation of instruments and equipment, 24-h esophageal pH monitoring was conducted in our study instead of ambulatory pH-impedance monitoring which currently recognized as gold standard for detection of reflux. As a result, weakly acidic (4 ≤ pH < 7), weakly alkaline (pH ≥ 7), gaseous, and re-reflux episodes could not be detected during the monitoring, which definitely decreased diagnostic yield of reflux monitoring in patients with atypical GERD [23].

## Conclusion

This retrospective study suggests no significant differences between 180° ARMS and 270° ARMS in rGERD patients regarding reflux control, relief of GERD symptoms, improvements on quality of life, and objective GERD parameters. However, the remarkable efficacy has been noted in relief of symptoms between pre- and post-ARMS treatment. According to our present findings, 180° ARMS might be more recommended for treatment of rGERD owing to lower incidence of newly postoperative dysphagia. However, balance between effective control of reflux and prevention of dysphagia needs to be further investigated.

## Supplementary Information

Below is the link to the electronic supplementary material.

Supplementary file1 (DOCX 20 KB)
