# Supplementary material for: Comparison of 180° anti-reflux mucosectomy versus 270° anti-reflux mucosectomy for treatment of refractory gastroesophageal reflux disease: a retrospective study
Source: Surg Endosc. 2021 Nov 15;36(7):5002–10. doi: 10.1007/s00464-021-08857-5 (PMC9160125; doi:10.1007/s00464-021-08857-5)
Supplement: Supplementary file 1 — Supplementary file1 (DOCX 20 KB) [file 464_2021_8857_MOESM1_ESM.docx]

**Supplementary Table 1. Patient inclusion and exclusion criteria**

| **Inclusion criteria:** |
| --- |
| • Patients who were aged 18 to 75 years |
| • Patients had gastroesophageal reflux disease (GERD) for at least 6 months |
| • Patients with persistent and troublesome reflux symptoms despite at least 8 weeks of a standard dose of proton pump inhibitors (PPIs)  • Patients with pathological esophageal acid exposure based on 24-h esophageal pH monitoring |
| • Signed written informed consent |
|  |
| **Exclusion criteria:** |
| • Patients with medical contraindications for upper endoscopy or general anesthesia |
| • Patients with coagulation disorders |
| • Patients with a body mass index > 35 (BMI: the weight in kilograms divided by the square of the height in meters) |
| • Patients with a hiatal hernia > 2 cm |
| • Patients with esophageal motility disorders |
| • Patients with severe active esophagitis (Los Angeles grade C or D) |
| • Patients with previous surgery or endoscopic treatment of the stomach or esophagus |
| • Pregnancy or breast feeding |
| • Lack of written and informed consent |
|  |

**Supplementary Table 2 Gastroesophageal Reflux Disease Questionnaire (GERD-Q)**

| **Questions** | **Frequency score (points) for symptom** | | | |
| --- | --- | --- | --- | --- |
|  | **0 day** | **1 day** | **2-3 days** | **4-7 days** |
| 1.How often did you have a burning feeling behind your breastbone (heartburn)? | 0 | 1 | 2 | 3 |
| 2.How often did you have stomach contents (liquid or food) moving upwards to your throat or mouth (regurgitation)? | 0 | 1 | 2 | 3 |
| 3. How often did you have pain in the center of the upper stomach? | 3 | 2 | 1 | 0 |
| 4. How often did you have nausea? | 3 | 2 | 1 | 0 |
| 5. How often did you have difficulty getting a good night's sleep because of your heartburn and/or regurgitation? | 0 | 1 | 2 | 3 |
| 6.How often did you take additional medication for your heartburn and/or regurgitation, other than what the physician told you to take) (such as Tums, Rolaids, Maalox?) | 0 | 1 | 2 | 3 |

**Supplementary Table 3. The GERD-HRQL scale**

| • Scale: No symptoms = 0; Symptoms noticeable, but not bothersome = 1; Symptoms noticeable and bothersome, but not every day = 2; Symptoms bothersome every day = 3; Symptoms affect daily activities = 4; Symptoms are incapacitating, unable to do daily activities = 5 | | | | | | |
| --- | --- | --- | --- | --- | --- | --- |
| • Questions | | | | | | |
| 1. How bad is your heartburn? | 0 | 1 | 2 | 3 | 4 | 5 |
| 2. Heartburn when lying down? | 0 | 1 | 2 | 3 | 4 | 5 |
| 3. Heartburn when standing up? | 0 | 1 | 2 | 3 | 4 | 5 |
| 4. Heartburn after meals? | 0 | 1 | 2 | 3 | 4 | 5 |
| 5. Does heartburn change your diet? | 0 | 1 | 2 | 3 | 4 | 5 |
| 6. Does heartburn wake you from sleep? | 0 | 1 | 2 | 3 | 4 | 5 |
| 7. Do you have difficulty swallowing? | 0 | 1 | 2 | 3 | 4 | 5 |
| 8. Do you have pain with swallowing? | 0 | 1 | 2 | 3 | 4 | 5 |
| 9. Do you have bloating or gassy feelings? | 0 | 1 | 2 | 3 | 4 | 5 |
| 10. If you take medication, does this affect your daily life? | 0 | 1 | 2 | 3 | 4 | 5 |
| How satisfied are you with your present condition? | Satisfied | | Neutral | | Dissatisfied | |

GERD-HRQL, gastroesophageal reflux disease-health related quality of life
